# Cardiorespiratory, Metabolic and Muscular Responses during a Video-Recorded Aerobic Dance Session on an Air Dissipation Platform

**DOI:** 10.3390/ijerph17249511

**Published:** 2020-12-18

**Authors:** Alessandra Moreira-Reis, José Luis Maté-Muñoz, Juan Hernández-Lougedo, Pablo García-Fernández, Eulogio Pleguezuelos-Cobo, Teresa Carbonell, Norma Alva, Manuel Vicente Garnacho-Castaño

**Affiliations:** 1 Department of Cell Biology, Physiology and Immunology, Faculty of Biology, University of Barcelona, 08028 Barcelona, Spain; alereis55@hotmail.com (A.M.-R.); tcarbonell@ub.edu (T.C.); nvalva@ub.edu (N.A.); 2 Laboratory of Biomechanics and Exercise Physiology, Department of Physical Activity and Sports Science, Alfonso X El Sabio University, 28691 Madrid, Spain; jmatmuo@uax.es (J.L.M.-M.); juanherlou@hotmail.com (J.H.-L.); 3 Department of Radiology, Rehabilitation and Physiotherapy, Complutense University of Madrid, 28040 Madrid, Spain; pablga25@ucm.es; 4 Physical Medicine and Rehabilitation Department, Hospital de Mataró, 08304 Mataró, Spain; epleguezuelos@csdm.cat; 5 TecnoCampus, GRI-AFIRS, School of Health Sciences, Pompeu Fabra University, 08302 Mataró, Spain

**Keywords:** ventilatory threshold, cardiopulmonary exercise test, fitness class, blood lactate, fatigue

## Abstract

Background: Aerobic dance (AD) is an appropriate physical activity for improving cardiorespiratory fitness. This study aimed to compare cardiorespiratory and metabolic responses, and muscle fatigue between an air dissipation platform (ADP) and a hard surface during a video-recorded AD session. Methods: 25 healthy young women (23.3 ± 2.5 years) completed three sessions. In session 1, participants performed an incremental test to exhaustion on a treadmill. One week after session 1, participants were randomly assigned in a crossover design to perform video-recorded AD sessions on an ADP and on a hard surface (sessions 2 and 3). Cardiorespiratory and metabolic responses were assessed during AD sessions. Muscular fatigue was measured before and after AD sessions by a countermovement jump test. Results: Significantly higher heart rate, respiratory exchange ratio, pulmonary ventilation, ventilatory oxygen equivalent, and ventilatory carbon dioxide equivalent were observed on an ADP than on a hard surface (*p* < 0.05). Despite a significant increase in lactate levels on an ADP (*p* ≤ 0.01), muscular fatigue and perceived exertion rating were similar on both surfaces (*p* > 0.05). Conclusions: Video-recorded AD on an ADP increased the cardioventilatory and metabolic responses compared to a hard surface, preventing further muscle fatigue.

## 1. Introduction

Group classes in fitness centers are a very popular physical activity among women and, particularly, aerobic dance (AD) is one of the most practiced worldwide. In this period of pandemic due to severe acute respiratory syndrome coronavirus type 2 (SARS-CoV-2), thousands of practitioners who performed AD classes in fitness centers have stopped training due to conditions of confinement. EHealth and exercise videos, television and mobiles are technologies that could be used to maintain physical function and mental health [1] during periods of confinement. AD led by fitness instructors through a video-recorded session could be a very interesting alternative to maintain or improve cardiorespiratory and metabolic fitness during periods of confinement.

Heart rate (HR), blood lactate levels and oxygen uptake (VO_2_) have been used as measurement parameters to assess the exercise intensity in AD classes [2]. De Angelis et al. proved that AD increased HR, VO_2_ and blood lactate concentrations to a greater extent than expected, showing a high exercise intensity, metabolic demand and a non-steady state [2]. The cardiorespiratory and metabolic requirements for regular bipedal work are conditioned by the mechanical properties of the surface [3,4]. Hardin et al. [3] concluded that a harder surface decreased VO_2_ compared to a softer surface. Rodrigues et al. demonstrated that an elastic surface (i.e., mini-trampoline) increased cardiovascular responses compared to a hardwood surface during a stationary running [5]. The authors assumed that the higher physiological demands induced by the mini-trampoline could be due to constant rebounds and instability produced by an elastic surface. This increased physiological demand would involve greater effort to carry out the exercise and maintain balance on the mini-trampoline. Moreover, soft surfaces may reduce the risk of joint injuries from high impact [6]. The physiological demands of AD classes could depend, at least in part, on the exercise intensity (i.e., percentage of maximal heart rate, percentage of maximal VO_2_, energy expenditure, blood lactate, etc.) and the type of surface.

The type of surface has been demonstrated to affect muscle fatigue [7], mechanical work [8] and energy cost [9]. Several studies have proposed that a compliant elastic surface reduces mechanical work and energy cost of generating muscle force compared with hopping or running on a hard surface [8,9]. In human motor actions, the energy cost is determined by the energy required to generate muscle force and the energy required to perform mechanical work [10,11]. During human hopping on a compliant elastic surface, part of the mechanical work is supplied by the musculoskeletal system. Another important part is provided by storage and recovery of elastic energy in the surface. As a consequence of increasing leg stiffness on a compliant elastic surface, the mechanical work done by the surface is increased. In contrast, the mechanical work done by the legs is reduced. Consequently, the energy cost is reduced by generating muscle force [9]. It is tempting to speculate that the different mechanical work and energy costs induced by different surfaces on the musculoskeletal system could lead to variations in muscular fatigue. Several studies have used vertical jump height (i.e., counter movement jump) before and after exercise to assess the extent of muscular fatigue [12,13]. However, muscular fatigue assessed by a countermovement jump test before and after an AD session has not been explored by comparing an ADP and a hard surface.

Recently, an air dissipation platform (ADP) has been incorporated by our research group into AD classes. The ADP consists of an area that rests on an elastomer that contains air and that allows air to enter and exit through holes. One of the main characteristics of this device is instability and rebound damping produced during exercise, just as it occurs on a mini-trampoline [5]. In theory, cardiorespiratory and metabolic responses on an ADP should be increased compared to a hard surface; however, this statement has not yet been scientifically confirmed. This knowledge would be a key factor in determining whether the exercise intensity during a video-recorded AD class on an ADP is enough to produce improvements in cardiorespiratory fitness.

This study aimed to assess the acute cardiorespiratory and metabolic responses induced by an ADP and a hard surface (marble floor) during a video-recorded AD session. The secondary aim was to determine the muscular fatigue induced by an ADP and a hard surface as well as the rate of perceived exertion (RPE). We hypothesized that a video-recorded AD session on an ADP produces higher acute cardiorespiratory and metabolic responses (blood lactate) compared to AD on a harder surface. In addition, a video-recorded AD session on an ADP is probably an ideal alternative to increase exercise intensity, maintaining similar RPE and muscular fatigue.

## 2. Materials and Methods

### 2.1. Experimental Approach to the Problem

Participants completed three test sessions at the Exercise Physiology laboratory. Sessions were conducted under the same environmental conditions (temperature 20–22.5 °C, atmospheric pressure: 715–730 mm Hg, and relative humidity 40–50%) and in the same time frame (+1 h). Participants refrained from any high-intensity physical effort for 48 h and abstained from any type of physical exercise for 24 h before starting the first session.

In session 1, an incremental test until exhaustion was completed on a treadmill to determine cardiorespiratory responses and ventilatory thresholds (VTs). One week after session 1, participants were randomly assigned in a crossover design to carry out AD sessions on an ADP and on a hard surface (session 2 and 3). The AD class was video recorded by a certified fitness instructor a week before. This video session was projected on a giant screen individually to each participant during the AD classes on an ADP or a marble floor (hard surface). Sessions 2 and 3 were rigorously identical and cardiorespiratory and metabolic responses, muscular fatigue and RPE were evaluated one week apart.

### 2.2. Participants

The participants recruited were 25 healthy young women (age, 23.3 ± 2.5 years; weight, 58.4 ± 6.8 kg; height, 162.6 ± 5.5 cm; and body mass index, 22.1 ± 2.4 kg/m^2^). All of them performed light or moderate physical activity a maximum of 2–3 times per week. Exclusion criteria were (a) the use of any medication or performance-enhancing drugs, (b) smoking or alcohol intake, (c) the intake of any nutritional supplement that could alter cardiorespiratory performance, (d) any cardiovascular, metabolic, neurological, pulmonary, or orthopedic disorders that could limit exercise performance, (e) being an elite athlete. Participants were informed of all experimental tests and signed an informed consent form. The study protocol received approval from the Ethics Committee of the University (13/2018) and adhered to the tenets of the Declaration of Helsinki.

### 2.3. Incremental Treadmill Test

The incremental cardiopulmonary exercise test (CPET) until exhaustion included a 5-min warm-up on a motorized treadmill (TechnoGym, Runrace 1400HC, Forlí, Italy) at a self-selected light intensity (~5–6 km·h^−1^), followed by 5-min of dynamic joint mobility drills and stretching exercises. After 3-min rest time, the CPET on a treadmill commenced at an initial load of 5 km·h^−1^ (1% slope) which was increased in steps of 0.5 km·h^−1^ every 30 s.

Respiratory exchange data were recorded during the CPET using a breath-by-breath open-circuit gas analyzer (Vmax spectra 29, Sensormedics Corp., Yorba Linda, CA, USA). VO_2_max, minute ventilation (VE), carbon dioxide production (VCO_2_), ventilatory equivalent for oxygen (VE·VO_2_^−1^), ventilatory equivalent for carbon dioxide (VE·VCO_2_^−1^), respiratory exchange ratio (RER), oxygen partial pressure on expiration (PetO_2_), partial pressure of carbon dioxide on expiration (PetCO_2_) were monitored. HR was checked every 5 s by telemetry (RS-800CX, Polar Electro OY, Kempele, Finland).

In the CPET, maximum or peak cardiorespiratory indices and VTs (first ventilatory threshold: VT1 and second ventilatory threshold: VT2) were determined to identify the relative exercise intensity of AD classes. As in a previous study [14], two investigators separately identified VT1 and VT2. If there was lack of agreement, the opinion of a third observer was considered. VT1 was defined as the workload (velocity) at which both VE·VO_2_^−1^ and PetO_2_ increase, without a concomitant increase in VE∙VCO_2_^−1^. Similarly, VT2 was defined as the workload (velocity) at which VE∙VO_2_^−1^ and VE∙VCO_2_^−1^ increase, accompanied by a drop in PetCO_2_ [15].

### 2.4. Aerobic Dance Sessions

AD sessions were conducted on an ADP and on a marble floor (hard surface) (sessions 2 and 3). The ADP consists of an area of one meter in diameter and 20 cm high that rests on an elastomer that contains air at atmospheric pressure and that allows air to enter and exit through holes. The same general warm-up was carried out as in the CPET. After a 3-min rest period, each subject performed a 40-min AD session of individual exercise on an ADP or a marble floor. The AD class consisted of three phases: a 5-min of specific warm-up, a 30-min aerobic or principal phase, and 5-min cool-down.

The aerobic phase of the AD session was structured by an experienced instructor to be of light intensity at most 75% HRmax (RPE ~11–12), moderate intensity at most 85% HRmax (RPE ~13–14), or heavy intensity at most 90% HRmax (RPE ~15–17) [16]. The AD sessions were based on global and multi-articular movements in which large muscle groups participated, including jumps, arm and leg movements, trunk flexions, etc. The exercise intensity of the AD classes was controlled by varying the muscle mass involved (deeper movements, increased bending, arm activity) as well as modifying the direction, the impact of the movements and the range.

**Figure 1:**
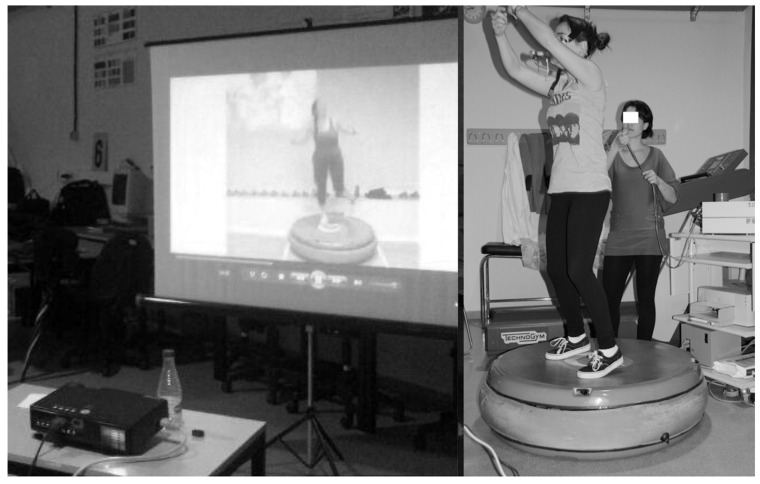
Aerobic dance session performed on an air dissipation platform. The video session was projected on a giant screen individually to each participant.

To verify that both AD classes (ADP vs. hard surface) were rigorously the same, a video of an AD class was recorded a week before. Participants were instructed to imitate the motor tasks to be performed by an expert instructor to the rhythm of the music (Figure 1). Since all participants were familiarized with AD classes, the motor tasks were not difficult to replicate.

### 2.5. Cardiorespiratory, Metabolic and Muscular Assessment

Respiratory exchange data were recorded during AD classes using a breath-by-breath open-circuit gas analyzer, as previously in the CPET.

Blood lactate and RPE were measured at rest (before warm-up) and every 10 min during AD classes (10-min, 20-min, 30-min and 40-min). Blood lactate levels were determined from finger capillary blood using a portable lactate analyzer (Lactate Pro LT-1710, Arkray Factory Inc., KDK Corporation, Siga, Japan), while RPE was determined by using the Borg Scale [17].

Before and after AD classes, muscular fatigue of lower limbs was evaluated by the countermovement jump (CMJ) test using a force platform (Quattro Jump model 9290AD; Kistler Instruments, Winterthur, Switzerland), as in previous studies [13,18]. The CMJ was initiated while standing on the force platform with hands on hips and legs extended. Next, the knees were first flexed to 90° (eccentric action) and immediately explosively extended in a coordinated manner (concentric action) trying to reach maximum vertical height. During the flight stage, the knees were fully extended and contact with the ground was made with the toes first. The participants were instructed to keep their hands on the hips and avoid any sideways or backward/forward movements during the flight stage.

Participants carried out 3 CMJs separated by a rest time of 30 s, and the mean values of vertical flight height and mean power (3 CMJs) were used in the subsequent analyses. Loss of vertical jump height and power output have been used to assess muscle fatigue before and after an exercise session [13,18]. The force platform was connected to a computer and the software package of Kistler (Quattro Jump software, version 1.1.1.4, (Kistler Instruments, Winterthur, Switzerland) was used to quantify the kinetic and kinematic variables. The vertical ground reaction force (GRF) data were obtained during the jump (range 0–10 kN; sampling frequency 0.5 kHz). The vertical component of the center of mass (COM) velocity was estimated using the impulse method [19]. Net impulse was taken by integrating the GRF from 2 s before the first movement of the participant [20]. The vertical velocity of COM was calculated by dividing the net impulse by the participant’s body mass [21]. Maximum velocity reached at the end of the concentric muscle action of the jump was considered as maximum take-off velocity (V_max_). Flight height (cm) was calculated from V_max_ of the COM and the deceleration of gravity. Height = ((V_max_)^2^/2 × 9.81). Power was calculated from the unfiltered force–time history using the impulse momentum principle [22]. Mean relative power (watts·kg^−1^) was calculated as the product of mean velocity and vertical component of the vertical ground reaction force.

### 2.6. Statistical Analysis

The Shapiro–Wilk test was used to check the normal distribution of data, provided as means, standard deviation (SD), confidence intervals (95% CI) and percentages. A t-student for paired samples was applied to identify significant differences between an ADP and a hard surface in cardiorespiratory and metabolic responses. Cohen’s d effect sizes (d < 0.4, small; ≥0.4, moderate; ≥0.8, large) were calculated to assess the magnitude of difference among experimental conditions [23].

A general linear model with a two-way analysis of variance (ANOVA) for repeated measures was performed to verify significant differences between an ADP and a marble hard surface in lactate levels and RPE. The two factors were exercise mode (ADP and marble floor) and time (corresponding to 4 checkpoints performed in both AD classes). When appropriate, a Bonferroni post hoc adjustment for multiple comparisons was implemented. An ANOVA for repeated measures was performed to determine muscular fatigue. The partial eta-squared (η_p_^2^) was computed to determine the magnitude of the response to both exercise modes. The statistical power (SP) was also calculated. All statistical methods were performed using the software package SPSS Statistics version 25.0 for Mackintosh (SPSS, Chicago, IL, USA). Significance was set at *p* < 0.05.

## 3. Results

Descriptive data related to the CPET in treadmill are presented in Table 1. The predicted maximum heart rate (197 beats·min^−1^) of the experimental group was not reached (187.1 ± 8.1 beats·min^−1^). The functional capacity of the healthy young women was good (VO_2_: 42.4 ± 7.5).

The differences among the experimental conditions are shown in Table 2. Significant higher acute responses in HR (*p* = 0.002, *t* = 3.5, moderate effect d = 0.4), RER (*p* = 0.031, *t* = 2.3, moderate effect d = 0.4), VE (*p* = 0.026, *t* = 2.4, small effect d = 0.3), VE·VO_2_^−1^ (*p* = 0.001, *t* = 3.7, moderate effect d = 0.5) and VE·VCO_2_^−1^ (*p* = 0.039, *t* = 2.2, small effect d = 0.2) were found on an ADP compared with a hard surface (marble floor). No significant differences were detected in the rest of the acute cardiorespiratory responses among experimental conditions (*p* > 0.05).

**Figure 2:**
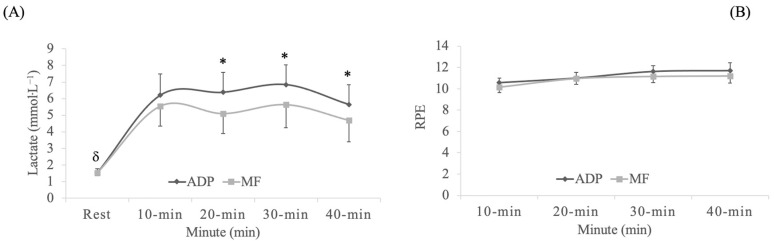
Blood lactate levels (**A**) and rating of perceived exertion (RPE) (**B**) during aerobic dance sessions. * Significantly different from marble floor at times 20-min, 30-min and 40-min, *p* ≤ 0.01. Data are provided as mean and 95% confidence intervals (95% CI). ^δ^ Significantly different from checkpoint at times10-min, 20-min, 30-min and 40-min in both experimental conditions, *p* < 0.001. Abbreviations used: ADP = air dissipation platform; MF = marble floor (hard surface).

In blood lactate concentrations, a significant exercise mode x time interaction effect was observed (*p* = 0.024, F_(4,88)_ = 2.9, η_p_^2^ = 0.1, SP = 0.8). A significant time effect was observed (*p* < 0.001, F_(4,88)_ = 44.0, η_p_^2^ = 0.7, SP = 1.0), and also a exercise mode effect (*p* = 0.002, F_(1,22)_ = 12.5, η_p_^2^ = 0.4, SP = 0.9). The Bonferroni test confirmed higher blood lactate levels when exercising on an ADP than a hard surface at 20-min, 30-min and 40-min (*p* ≤ 0.01) (Figure 2A). RPE followed the same evolution in both exercise groups (*p* > 0.05) (Figure 2B).

**Figure 3:**
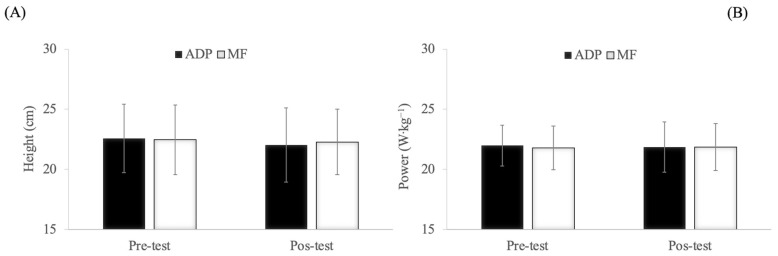
Muscular fatigue assessed by countermovement jump (CMJ) test before and after the aerobic dance classes. (**A**) Mean height (cm) in CMJ. (**B**) Mean power (W·kg^−1^) in CMJ. No significant differences were detected among experimental conditions. Abbreviations used: ADP = air dissipation platform; MF = marble floor (hard surface).

In the CMJ test, no significant exercise mode × time interaction effect, time and exercise mode effects were observed (*p* > 0.05) (Figure 3).

## 4. Discussion

The main finding of this study was that AD led by fitness instructors through a video-recorded session induced a higher HR, VE, RER, VE·VO_2_^−1^, VE·VCO_2_^−1^ and blood lactate concentrations on an ADP than on a hard surface. AD performed on both surfaces induced similar muscular fatigue in the lower extremities and feeling of physical exertion (RPE).

The HR was greater on an ADP (+3.8%) than on a hard surface. Recently, Rodrigues et al. [5] observed that elastic surfaces (mini-trampoline 76.5% of HRmax) increased the HR response compared to a hard surface (67.4% of HRmax) during a stationary running. It is possible that the higher HR observed on an ADP could be due to the rebounding and instability induced by the elastic surface, and consequently it would involve a greater effort to maintain balance during AD session [5]. AD in both surfaces indicated a percentage of peak HR similar to those reported in aerobic step dance performed with load (89.8%) [24]. However, the HR was higher on both surfaces than those observed in tap dance (83.8%) [25] and in an aerobic step dance performed without overload (84.5%) [24]. AD activities such as body pump, body combat and spinning have shown a low HR (60.2%, 73.2%, 74.3% of HRmax, respectively) [26]. Apart from the contact surface, the discrepancy in HR response could be attributed to the different dance modalities and class methodologies used for AD.

The observed HR in both surfaces demonstrated that a video-recorded AD session could be an adequate alternative for improving or maintaining cardiorespiratory fitness in healthy young women. The American College of Sports Medicine (ACSM) guidelines determines that vigorous intensity (≥~6 metabolic equivalents (METs), 77–95% of HRmax) of physical activity performed 3 d·wk^−1^ for a total of ~75 min·wk^−1^ or 20 min·d^−1^ improves or maintains cardiorespiratory fitness [27]. In this regard, both AD sessions may be considered as a vigorous exercise intensity (ADP: 8.6 MET/91.8% of HRmax vs. hard surface: 8.5 METs/88.6% of HRmax).

Blood lactate levels were increased to a greater extent on an ADP compared to a hard surface. Brito et al. [28] showed that unstable surfaces such as sand produced greater blood lactate levels in comparison to hard surfaces (4 mmol·L^−1^ and 2.8 mmol·L^−1^) during a soccer game. The authors concluded that playing on hard surfaces such as asphalt decreased the lactate anaerobic pathway more than playing on unstable surfaces. This appreciation suggests that differences in blood lactate levels between an ADP and a hard surface could be due to the fact that the impact forces induced by the damping are reduced on the platform. The instability and contact times on ADP would increase, causing increased muscle activation in the agonist and antagonist muscles as occurs on unstable surfaces [29]. This amplified muscle activation could accentuate the muscular work and, consequently, the lactate anaerobic metabolic pathway could be further activated. Future studies should be focused on comparing the activation of metabolic pathways between unstable and stable surfaces as long as the intensity of exercise proposed was the same.

In a similar study [25], lower blood lactate levels were found during a tap dance choreography compared to our study (1.7 mmol·L^−1^ vs. 6.3 mmol·L^−1^ and 5.2 mmol·L^−1^, respectively). We suggested that the differences observed between studies could be attributed to exercise intensity. The relative exercise intensity established in both AD activities was interpreted based on the ventilatory parameters obtained during an incremental treadmill test. To this end, VT1 and VT2 were determined by expert evaluators since it has been well established that performance in endurance exercise is linked to VTs [30,31]. To our knowledge, no studies have used VTs during an incremental treadmill test as a reference to identify the relative exercise intensity in an AD class performed on elastic and hard surfaces. Nevertheless, Oliveira et al. [25] used the lactate threshold in an incremental treadmill test to categorize the exercise intensity during a tap dance choreography. The lactate threshold and VT1 are certainly connected and occur at a comparable exercise intensity in several forms of exercise [32,33]. This disagreement among studies could be supported by the fact that tap dance exercise intensity was approximately 10% less than the lactate threshold. Our outcomes showed a relative exercise intensity above VT1 and next to VT2 in both surfaces. Accordingly, higher HR (ADP 8.7%, marble floor 5.4%), VO_2_ (ADP 2.8%, marble floor 2.1%) and METs (ADP 5.8%, marble floor 4.7%) were observed in both AD sessions than in a tap dance choreography [25]. This augmented cardiorespiratory response justified, at least in part, a greater blood lactate levels observed in our study.

These differences in blood lactate concentrations among studies highlight the relevance of previously knowing exercise intensity (HR) through check points (ventilatory or lactate thresholds) that determine the metabolic changes in each participant. In this way, practitioners of activities such as fitness classes would know the workload or relative intensity (HR or VO_2_) corresponding to the session. Additionally, researchers would be able to understand the underlying adaptive physiological mechanisms of the various AD modalities.

It is clear that knowledge of these VTs has been a key factor in discovering the cardiorespiratory exercise intensity and, consequently, understanding why there was a greater metabolic stress than in other studies. Ventilatory adaptation to CO_2_ production threshold (VT1) is interpreted as the first non-linear increases in VCO_2_ and ventilation due to the bicarbonate buffering of H^+^ produced by a gradual increase in blood lactate levels above resting values [34]. VT2 is accepted as the second breakpoint in the ventilation response chiefly produced by a pH decrease as bicarbonate is saturated by the rising production of lactate (acidosis) [35]. Probably, the higher VE, RER, VE·VO_2_^−1^, VE·VCO_2_^−1^ detected on an ADP than on a marble floor could be due to this increase in acidosis and relative intensity very close to that observed during the incremental treadmill test in VT2.

In theory, this increased cardiac and metabolic stress would imply a higher VO_2_ on an ADP than on a marble floor. However, VO_2_ was similar among surfaces. VO_2_ results differed from others that showed how elastic surfaces increased HR and VO_2_ to a larger magnitude than hard surface [5]. Although we have not found studies evaluating several surfaces in AD modalities, a study on competitive tennis players revealed that the HR and blood lactate levels were higher on a softer surface, maintaining similar VO_2_ [36]. Given these discrepancies, we did not find a rational physiological and biomechanical explanation. More studies are necessary to draw adequate conclusions.

VO_2_ was similar in both surfaces (ADP: 70.9% vs. hard surface 70.4% of VO_2_max). Analogous findings were found during stationary running on an elastic surface (68.9% of VO_2_max) and aerobic step dance performed without overload (68.9% of VO_2_max) confirming an adequate relative exercise intensity to provide cardiovascular improvements [25]. Nevertheless, VO_2_ (78.3% of VO_2_max) was increased in aerobic step dance carried out with overload [24]. External overload could be an added resource to increase the relative intensity (% of HRmax and VO_2_max) produced by the rebound effect and the instabilities of the elastic surfaces.

Muscular fatigue has been assessed by CMJ test in several studies that analyzed cardiorespiratory and metabolic responses [12,13,18]. Despite finding a greater cardiometabolic and ventilatory stress on an ADP than on a hard surface, mechanical fatigue on an ADP was not augmented compared to a hard surface. We suspect that an ADP reduced the mechanical work done by the lower limbs by increasing leg stiffness on compliant elastic surface. Our arguments cannot be objectively corroborated since leg stiffness on an ADP was not measured, thus these interpretations remain purely speculative. However, it is assumed that the mechanical work is reduced by increasing leg stiffness on a compliant elastic surface [8,9]. Therefore, the joint strain and muscle fatigue could be minimized by using elastic surfaces. Soft surfaces may reduce the risk of joint injuries at high impact [6].

It would be logical to identify a greater perception of effort in response to a higher cardioventilatory and metabolic stress on an ADP; however, the RPE of the participants was the same throughout the session. The RPE is an excellent parameter to subjectively quantify the exercise intensity in fitness sessions [37]. It was the first time that participants used an ADP; therefore, the motivation to use a different apparatus could have been a differential factor for having a lesser sensation of physical effort. The greater feeling of pleasure on an ADP than on the marble floor was verified by the participants. More research would be necessary to determine the psychological and emotional benefits and adherence that this type of device could produce.

An AD session on an ADP could increase cardiorespiratory and metabolic demands by diminishing mechanical stress on the joints and muscle mass. Thus, this type of aerobic activity could be very suitable for elderly people (skeletal fragility) because they could achieve the same functional exercise intensity with less mechanical stress. Determining exercise intensity based on the type of surface in an AD class by using VTs would allow us to know whether acute cardioventilatory and metabolic responses are intense enough to promote long-term improvements (adaptations) in cardiorespiratory fitness.

Finally, the findings of this study are relevant because a video-recorded AD session on an ADP could be an excellent alternative to perform physical activity during forced periods of confinement due to pandemics (i.e., SARS-CoV-2). Long stays at home are likely to encourage sedentary behaviors such as spending more time sitting, lying down, playing video games, etc., leading to an increased risk and potential worsening of chronic health conditions. In this environment, the practice of physical activity at home is more than justified. Performing safe, simple and easy to implement exercises at home is very suitable to avoid airborne coronavirus and maintain fitness levels [38]. The ADP is a safe, simple and small device that can be used at home. The findings reported in this study demonstrated that a video-recorded AD session on an ADP induced a suitable exercise intensity. The supervision of fitness instructors would be recommended during AD sessions on an ADP to establish adequate guidelines for improving cardiorespiratory fitness in exercise home-programs. AD on an ADP performed 3 d·wk^−1^ for a total of ~75 min·wk^−1^ or 20 min·d^−1^ could be a very interesting alternative to maintain or improve cardiorespiratory and metabolic fitness according to the ACSM guidelines.

## 5. Conclusions

A video-recorded AD class on an ADP increases greater cardiorespiratory and metabolic responses than on a harder surface, without inducing greater muscular fatigue and feeling of physical exertion.

## Figures and Tables

**Table 1:** Cardiorespiratory results attained during the incremental treadmill test.

Variables	Mean (SD)
Peak HR (beats·min^−1^)	187.1 (8.1)
Peak VO_2_ (L·min^−1^)	2.4 (0.5)
Peak VO_2_ (mL·kg^−1^·min^−1^)	42.4 (7.5)
Peak VCO_2_ (L·min^−1^)	2.9 (0.5)
Peak RER	1.2 (0.1)
Peak VE (L·min^−1^)	84.5 (15.9)
Peak Velocity (km·h^−1^)	12.0 (1.6)
HR at VT_1_ (beats·min^−1^)	152.2 (15.1)
HR at VT_1_ (%)	81.3 (7.2)
VO_2_ at VT_1_ (L·min^−1^)	1.5 (0.5)
VO_2_ at VT_1_ (mL·kg^−1^·min^−1^)	26.0 (7.3)
VO_2_ at VT_1_ (%)	61.3 (11.2)
VCO_2_ at VT_1_ (L·min^−1^)	1.3 (0.4)
RER at VT_1_	0.9 (0.1)
VE at VT_1_(L·min^−1^)	37.4 (10.4)
Velocity at VT_1_ (km·h^−1^)	7.1 (1.1)
HR at VT_2_ (beats·min^−1^)	174.8 (12.2)
HR at VT_2_ (%)	93.4 (6.1)
VO_2_ at VT_2_ (L·min^−1^)	2.2 (0.5)
VO_2_ at VT_2_ (mL·kg^−1^·min^−1^)	36.9 (7.0)
VO_2_ at VT_2_ (%)	87.0 (6.7)
VCO_2_ at VT_2_ (L·min^−1^)	2.2 (0.5)
RER at VT_2_	1.0 (0.1)
VE at VT_2_ (L·min^−1^)	61.2 (14.6)
Velocity at VT_2_ (km·h^−1^)	9.8 (1.4)

Abbreviations used: HR = heart rate; MET = metabolic equivalent; RER = respiratory exchange ratio; standard deviation = SD; VCO_2_ = carbon dioxide production; VE = minute ventilation; VO_2_ = oxygen uptake; VT_1_ = first ventilatory threshold; VT_2_ = second ventilatory threshold. (n = 25).

**Table 2:** Acute cardiorespiratory responses during both experimental conditions.

Variables	ADP	% Peak Values ^λ^	MF	% Peak Values ^λ^
HR (beats·min^−1^)	171.7 (12.6) *	91.8	165.7 (14.6)	88.6
VO_2_ (L·min^−1^)	1.8 (0.3)	72.0	1.7 (0.3)	71.6
VO_2_ (mL·kg^−1^·min^−1^)	30.0 (3.5)	70.9	29.8 (3.7)	70.4
VCO_2_ (L·min^−1^)	1.7 (0.2)	59.0	1.6 (0.3)	57.4
RER	1.0 (0.1) ^δ^	81.2	0.9 (0.1)	79.4
VE (L·min^−1^)	59.0 (9.6) ^δ^	69.8	55.5 (11.6)	65.7
VE·VO_2_^−1^	34.1 (4.1) *	96.0	32.2 (3.7)	90.5
VE·VCO_2_^−1^	34.3 (3.2) ^δ^	100	33.4 (3.4)	100
METs	8.6 (1.4)	70.9	8.5 (1.1)	70.3

Data are presented as mean and standard deviation (SD). Abbreviations used: ADP = Air dissipation platform; HR = heart rate; MET = metabolic equivalent; MF = marble floor; RER = respiratory exchange ratio; VCO_2_ = carbon dioxide production; VE = minute ventilation; VE·VO_2_^−1^ = ventilatory equivalent for oxygen; VO_2_ = oxygen uptake. * Significantly different from MF, *p* < 0.01. ^δ^ Significantly different from MF, *p* < 0.05. ^λ^ percentage considers peak values obtained in the incremental treadmill test.
